# Cooperativity of Spin Crossover Complexes: Combining Periodic Density Functional Calculations and Monte Carlo Simulations

**DOI:** 10.3390/ma10020172

**Published:** 2017-02-13

**Authors:** Lars Kreutzburg, Christian G. Hübner, Hauke Paulsen

**Affiliations:** Institut für Physik, Universität zu Lübeck, Ratzeburger Allee 160, 23562 Lübeck, Germany; huebner@physik.uni-luebeck.de

**Keywords:** spin crossover, density functional calculations, Hubbard model, solid state, Slichter–Drickamer model, Monte Carlo simulation, Ising model, 71.15.Mb, 75.10.Hk, 75.30.Wx

## Abstract

The total enthalpies of the 16 different spin configurations that can be realized in the unit cell of the archetype spin crossover complex [Fe(phen)${}_{2}$(NCS)${}_{2}$] (phen = 1,2-phenanthroline) were calculated, applying periodic density functional theory combined with the Hubbard model and the Grimme-D2 dispersion correction (DFT+*U*+D2). The obtained enthalpy differences between the individual spin configurations were used to determine spin couplings of an Ising-like model, and subsequent Monte Carlo simulations for this model allowed the estimation of the phenomenological interaction parameter Γ of the Slichter–Drickamer model, which is commonly used to describe the cooperativity of the spin transition. The calculation procedure described here—which led to an estimate of about 3 kJ·mol${}^{-1}$ for Γ, in good agreement with experiment—may be used to predict from first principles how modifications of spin crossover complexes can change the character of the spin transition from gradual to abrupt and vice versa.

## 1. Introduction

**Figure 1:**
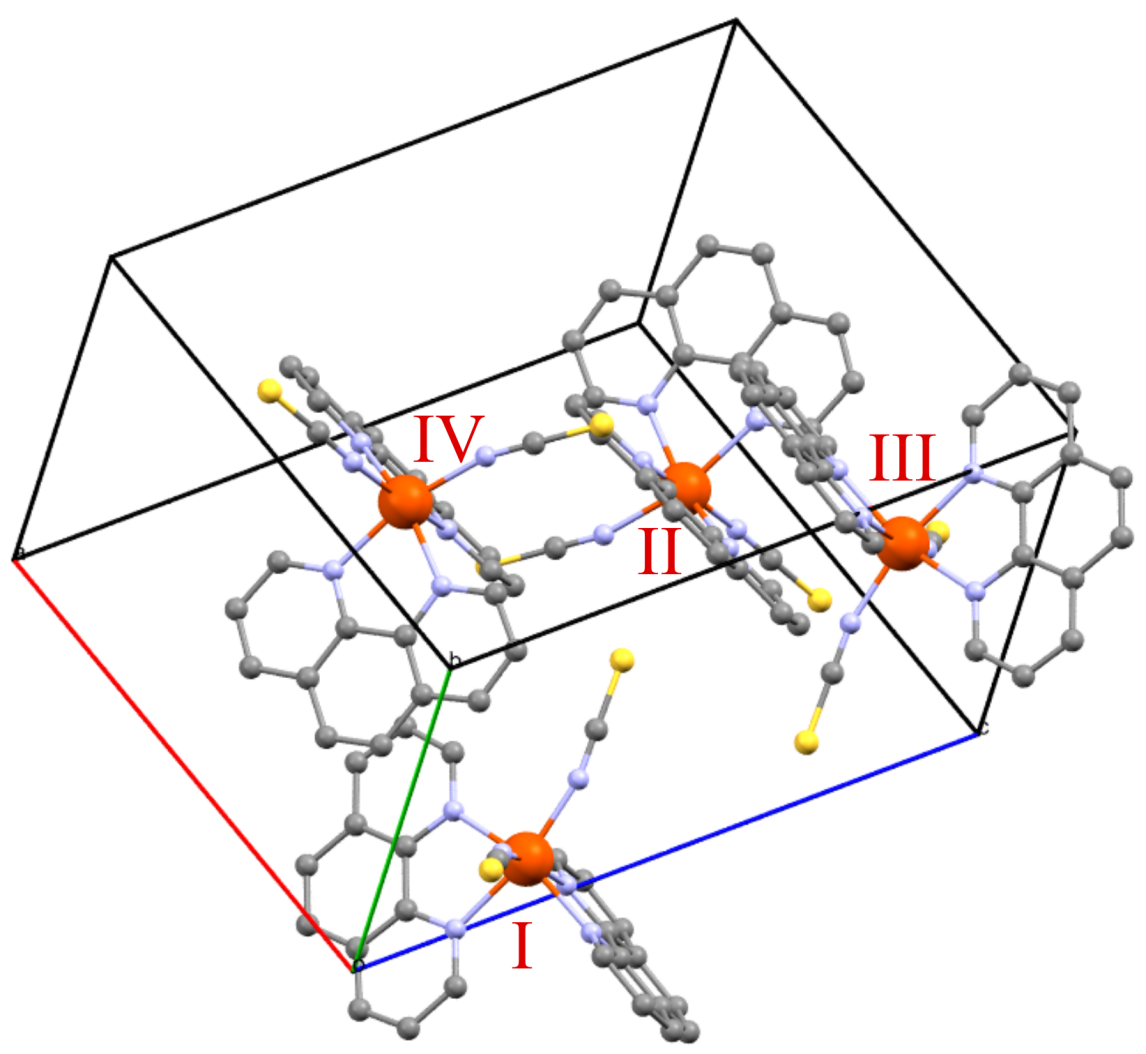
Calculated unit cell of the low spin (LS) phase of [Fe(phen)${}_{2}$(NCS)${}_{2}$] with four symmetry equivalent molecules labelled with Roman numbers (colour scheme: Fe (red), S (yellow), N (blue), and C (grey); hydrogens are omitted for clarity). Molecules I and IV are located in a sheet parallel to the a–b plane, a second parallel sheet contains molecules II and III.

Spin crossover (SCO) complexes [1,2,3,4] are a special class of transition metal compounds that exhibit a change of the electronic spin state from a high spin (HS) to a low spin (LS) state when the temperature is decreased or the external pressure is increased. SCO complexes (see Figure 1 for an example) are promising materials for memory devices with extremely high capacities, since some of them may be reversibly switched between metastable HS and LS states by irradiation with light—a phenomenon known as the LIESST effect (light induced spin state trapping) [5].

Alongside a richness of experimental studies [1,2,3,4], a large number of theoretical investigations on SCO systems have been performed, mostly in the past two decades (for review see, e.g., References [6,7,8,9]). While much work has been done to predict transition temperatures, far less effort has been put into calculating the character (gradual or abrupt) of the thermally-induced spin transition [10,11,12]. For technical applications, however, the abruptness of the transition and the width of the thermal hysteresis is as important as a suitable transition temperature.

### 1.1. Slichter–Drickamer Model

An easy and in the course of the years widespread theoretical model to describe the character of thermal spin transitions has been presented by Slichter and Drickamer [13]. This so-called Slichter–Drickamer model (SDM for short) belongs to the class of mean-field theories. It traces the cooperativity of the transition back to an interaction between individual spins and the average magnetization of the crystal. The strength of this interaction is given by the phenomenological interaction parameter Γ. If this macroscopic parameter is positive, a spin can decrease its energy by adopting the majority spin state, and the temperature dependent HS fraction $\gamma_{\mathrm{HS}}\left(T\right)$—the fraction of molecules that are in the HS state—will undergo an abrupt transition from about 0 to almost 1 at the transition temperature $T_{1/2}$, which is implicitly defined by $\gamma_{\mathrm{HS}}\left(T_{1/2}\right)=1/2$. On the other hand, a negative Γ corresponds to a more gradual transition. In the framework of the SDM, the Gibbs energy (or free enthalpy) per molecule can be written as

(1) $$G\left(\gamma_{\mathrm{HS}}\right)=\gamma_{\mathrm{HS}}\left(\Delta E_{\mathrm{HL}}-T\Delta S_{\mathrm{HL}}+p\Delta V\right)+\gamma_{\mathrm{HS}}\left(1-\gamma_{\mathrm{HS}}\right)\;\Gamma -TS_{\mathrm{mix}},$$

where $\Delta E_{\mathrm{HL}}$ and $\Delta S_{\mathrm{HL}}$ are the differences of the total electronic energy and entropy, respectively, between HS and LS states. The mixing entropy $S_{\mathrm{mix}}$ is defined by

(2) $$S_{\mathrm{mix}}=-k_{B}\left[\gamma_{\mathrm{HS}}\ln\gamma_{\mathrm{HS}}+\left(1-\gamma_{\mathrm{HS}}\right)\ln\left(1-\gamma_{\mathrm{HS}}\right)\right].$$

Since the contribution of volume expansion, pΔV, usually amounts to less than 1 J·mol${}^{-1}$ at ambient pressure [6], it can be safely neglected in comparison to $\Delta E_{\mathrm{HL}}$, which is four orders of magnitude larger. Therefore, where in the following the total energy difference is mentioned, it is for all practical purposes in the present study synonymous with the total enthalpy difference. For simplicity, the Gibbs energy of the pure LS phase has been arbitrarily set to zero (G(0)=0), which does not change the dynamics of the system that is solely determined by the differences of Gibbs energy. In the equilibrium, the variation of Gibbs energy with respect to $\gamma_{\mathrm{HS}}$ must vanish at any given temperature,

(3) $$\frac{\partial G}{\partial \gamma_{\mathrm{HS}}}=0,$$

a condition from which the well-known implicit equation for the temperature-dependent HS fraction [13],

(4) $$k_{B}T\ln\left(\frac{1-\gamma_{\mathrm{HS}}}{\gamma_{\mathrm{HS}}}\right)=\Delta E_{\mathrm{HL}}-T\Delta S_{\mathrm{HL}}+\left(1-2\gamma_{\mathrm{HS}}\right)\;\Gamma$$

can be derived.

This equation can be either used to determine parameters like $\Delta E_{\mathrm{HL}}$, $\Delta S_{\mathrm{HL}}$, and Γ by fitting a measured $\gamma_{\mathrm{HS}}\left(T\right)$ curve [11] or to predict such a curve by means of calculated parameters (e.g., from DFT calculations) [10,11,12].

### 1.2. Periodic Density Functional Calculations

**Figure 2:**
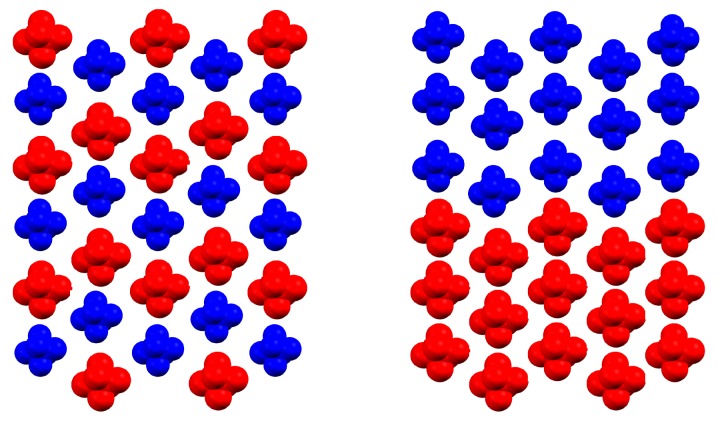
Two schematic depictions of a sheet parallel to the b–c plane of [Fe(phen)${}_{2}$(NCS)${}_{2}$], filled with high spin (HS) and low spin (LS) molecules (coloured in red and blue, respectively). Both configurations yield $\gamma_{\mathrm{HS}}=0.5$. The left configuration ($c_{\ell }$) corresponds to the unit cell configuration 6 in Table 1, while the right configuration ($c_{r}$) cannot be understood as a periodic continuation of a unit cell configuration.

While the SDM and Equation (1) refer to macroscopic samples, which may be reasonably well described by $\gamma_{\mathrm{HS}}$ alone, DFT calculations are necessarily restricted to microscopic systems (e.g., unit cells), the state of which has to be described by the configuration *c* of all individual spin states. Obviously, if the configuration *c* of a system is known, the HS fraction can uniquely be determined; on the other hand, a given HS fraction may originate from two very different configurations. For instance, $c_{\ell }$ and $c_{r}$ in Figure 2 correspond to the same HS fraction: $\gamma_{\mathrm{HS}}\left(c_{\ell }\right)=\gamma_{\mathrm{HS}}\left(c_{r}\right)$. Furthermore, in most cases, DFT calculations are used to obtain the electronic and geometrical structure at zero temperature. In this case, the entropic terms in Equation (1) vanish, and it is sufficient to consider the electronic energy instead of the Gibbs energy. The total electronic energy per molecule can thus be written as

(5) $$E\left(c\right)=\gamma_{\mathrm{HS}}\left(c\right)\Delta E_{HL}+\gamma_{\mathrm{HS}}\left(c\right)\left[1-\gamma_{\mathrm{HS}}\left(c\right)\right]\;\Gamma \left(c\right),$$

where a configuration-dependent auxiliary quantity Γ(c) has been introduced that is hereby defined as

(6) $$\Gamma \left(c\right)=\frac{E\left(c\right)-\gamma_{\mathrm{HS}}\Delta E_{\mathrm{HL}}}{\gamma_{\mathrm{HS}}\left(c\right)\left[1-\gamma_{\mathrm{HS}}\left(c\right)\right]}$$

for all possible configurations *c*, except the pure LS and HS configurations. These two configurations, however, are not relevant for estimating Γ, which can be done by multiplying Γ(c) with the Boltzmann weight of configuration *c* and summing up these terms for all possible *c*.

The first attempt to obtain Γ in this way from first principles calculations [12] yielded a value that was twice as large as the value derived experimentally. A possible explanation for this overestimation of Γ is based on the limited number of configurations that were used in Reference [12]. If only few configurations are accessible, only large values of E(c) on the right side of Equation (6) are contributing, and consequently, the interaction parameter Γ will be overestimated. For instance, the two configurations illustrated in Figure 2 both correspond to $\gamma_{\mathrm{HS}}=1/2$, but only the left could be assessed by DFT calculations for small systems. The right one instead—which may well be lower in energy and lead to a smaller Γ(c)—can only be assessed if the calculations are performed for large systems that are out of reach for DFT methods. Recently, hybrid Monte Carlo/molecular dynamics (MC/MD) calculations with ligand field force fields (LF FF) have been presented to overcome this problem [14,15,16]. An alternative approach using Ising-like models is presented in the next section.

### 1.3. Ising-Like Models

Since DFT calculations are restricted to microscopic systems containing no more than a few SCO molecules, and since several important features of the thermal spin transition in solid state SCO systems such as abruptness, hysteresis, or intermediate plateaus cannot be understood on the basis of microscopic models for single molecules or unit cells alone, it seems reasonable to combine DFT calculations with a method that is suitable for the simulation of larger systems. Ising-like models are auspicious candidates for such a method, and they have been introduced into the field of SCO research for more than two decades [17,18,19]; they now an established tool for the analysis of SCO phenomena (recent examples are given by [20] or [21]). Among these Ising-like models, there are sophisticated models to investigate various properties of SCO systems like width of hysteresis, time dependent dynamics, or surface effects.

For the purpose of the present study—the derivation of Γ from electronic structure calculations—a very basic model is sufficient, and can be defined as follows: a cubic supercell is constructed that consists of n×n×n unit cells of the SCO crystal of interest, where the integer *n* determines the side length of the cubic supercell. To each metal center of an SCO molecule labelled with index *i*, a spin $\sigma_{i}$ is assigned which can take values of +1 or −1 (if the metal center is in the HS or in the LS state, respectively).

The Hamiltonian for this systems of Ising spins is given by

(7) $$H=H_{0}-\sum_{i}\sigma_{i}B-\sum_{i,j}J_{i,j}\sigma_{i}\sigma_{j},$$

where the energy *B* has a formal similarity with an external field that is the same for each spin $\sigma_{i}$, and $J_{i,j}$ are coupling parameters that describe interactions between neighbouring spins $\sigma_{i}$ and $\sigma_{j}$. $H_{0}$ is a constant chosen in a way that the energy of the pure LS lattice is zero. With respect to Equation (1), the energy *B* can be written as

(8) $$B=-\frac{1}{2}\left(\Delta E_{HL}+T\Delta S_{HL}-p\Delta V\right).$$

In the simplest case of non-interacting molecules (all couplings $J_{i,j}$ are zero), an analytic solution for the thermal spin expectation value is available from textbooks:

(9) $$\langle \sigma \rangle =\tanh\left(B/k_{B}T\right),$$

which corresponds to a gradual spin transition. A simple way to include intermolecular interactions into the Ising-like model is the mean-field (mf) approximation, which is based on the assumption that each spin $\sigma_{i}$ only feels the averaged spin states of its neighbours. In this case, the generalized “external field” *B* in Equation (8) has to be augmented by a term

(10) $$B_{\mathrm{mf}}=\frac{\Gamma }{4}\langle \sigma \rangle$$

that is proportional to the spin expectation value. The Hamiltonian of the mean-field approximation can thus be written as

(11) $$H_{\mathrm{mf}}=H_{0}-\sum_{i}\sigma_{i}\left(B+B_{\mathrm{mf}}\right).$$

Monte Carlo simulations with this simplified Hamiltonian as well a self-consistent solution of the SDM Equation (4) are both mean-field approaches, and should yield the same transition curve. The purpose of the mean-field Hamiltonian for the Ising-like model is therefore the possibility to test the Monte Carlo simulations. This equivalence of MC simulations and SDM is of course only valid if the mean-field Hamiltonian (11) is used. In comparison to the original Hamiltonian (7) of the Ising model, the mean-field approach is an approximation that becomes exact only in the limiting case of vanishing cooperativity (that is, if the absolute values of the coupling parameters $J_{ij}$—or, equivalently—the SDM parameter Γ become zero.)

### 1.4. [Fe(phen)${}_{2}$(NCS)${}_{2}$]

The SCO complex [Fe(phen)${}_{2}$(NCS)${}_{2}$] with phen=1,2-phenanthroline (Figure 1) has been chosen as a complex that is most suitable to test the methods described above. It is certainly one of the most intensively studied SCO complexes, and also one of the very few that have been studied by periodic DFT calculations [12,22,23,24,25,26,27]. High-precision crystal structures are available for the HS and LS phases at different temperatures down to 15 K [28,29]. An abrupt transition from a S=0 low spin to a S=2 high spin state has been observed at about 176 K, together with a narrow thermal hysteresis. From the measured transition curve, Sinitskiy et al. [11] derived an interaction parameter Γ of about 3.0 kJ·mol${}^{-1}$ compared to a value of 7.3 kJ·mol${}^{-1}$ that has recently been estimated on the basis of periodic DFT calculations [12].

## 2. Results and Discussion

The strategy pursued in this study can be outlined as follows: in a first step, the total enthalpy of the unit cell of [Fe(phen)${}_{2}$(NCS)${}_{2}$] was obtained from periodic DFT calculations for all possible configurations of spin states. Subsequently, these results were used to determine coupling constants of an Ising-like model in a way that the energy levels of the DFT calculations were reproduced. Finally, Monte Carlo simulations for the Ising-like model were carried out for different lattices with increasing sizes in order to estimate the interaction parameter Γ.

### 2.1. DFT Calculations

Sixteen different spin configurations are possible for the four molecules of the unit cell of [Fe(phen)${}_{2}$(NCS)${}_{2}$]. These are labelled by a four letter code (see Table 1), where the letters L (low spin) and H (high spin) denote the spin states of the molecules in the order defined in Figure 1 (e.g., HLLL represents a configuration with molecule I in the HS state and molecules II, III, and IV in the LS state). Due to the symmetry of the space group Pbcn, only seven out of the sixteen configurations are independent [11], meaning that no more than seven different configurations can be found where no one can be transformed into any other one by symmetry operations. This implies, for instance, that all four configurations with only one HS molecule (nos. 2–5 in Table 1) should have the same energy, and the same is valid for the four configurations with three HS molecules (nos. 12–15). The six configurations with two HS and two LS molecules form three doubly-degenerate energy levels. Nevertheless, in order to test the accuracy of this approach, for each of the sixteen configurations, a complete variable-cell geometry optimization was performed using the gradient corrected density functional from Perdew, Burke and Ernzerhof (PBE) [30], and the total electronic energies per molecule, E(c), have been calculated (see Table 1). Previous DFT calculations employing the local density approximation (LDA) [12] reproduced only roughly the energetic degeneracy of symmetry equivalent configurations. It is assumed here that the cutoff of 50 Ry for the plane waves that was used in Reference [12] is too low in energy, so precision changes coming along with changes of the cell size during cell optimization produced artefacts [26]. The present calculations with a cutoff of 75 Ry have instead reproduced the degeneracy of equivalent configurations well.

The calculated volumes of the unit cell in the LS and HS phases (2124 Å${}^{3}$ for configuration LLLL and 2207 Å${}^{3}$ for HHHH)—both of which correspond to a temperature of 0 K—are in good agreement with the experimental values (2219 Å${}^{3}$ at 130 K and 2338 Å${}^{3}$ at 293 K—measured by Gallois et al. [28]; 2186 Å${}^{3}$ at 15 K and 2248 Å${}^{3}$ at the same temperature after irradiation with light—measured by Legrand et al. [29]), in contrast to previous LDA calculations, where good agreement could be reached only for the volume expansion upon spin transition [12], due to the notorious underestimation of bond lengths by LDA methods [31].

The proper choice of the Hubbard parameter *U* is obviously connected to the choice of the density functional. For calculations employing gradient-corrected functionals, values of about 2.5 eV are normally chosen for *U* [22,24,26], whereas larger *U* values were reported for LDA calculations [12,25]. It is known that electron correlation is less well accounted for by local density functionals than by gradient corrected ones, and therefore, LDA methods need a larger *U* in order to localize the strongly correlated iron 3d electrons. In the present study, the choice of U=2.5 eV led to a calculated electronic energy difference of $\Delta E_{\mathrm{HL}}\approx 10.3$ kJ·mol${}^{-1}$, which is in reasonable agreement with the experimentally determined 9 kJ·mol${}^{-1}$ from Sorai and Seki [32]. $\Delta E_{\mathrm{HL}}$ depends linearly and quite sensitively on the choice of *U*. An increase of *U* by less than 1% would decrease $\Delta E_{\mathrm{HL}}$ by more than 10% in the present case. However, the periodic DFT+*U*+D2 calculations applied here would lose their predictive power if better agreement between experimentally determined and theoretically calculated $\Delta E_{\mathrm{HL}}$ were to be achieved by fine tuning *U*.

In the absence of any cooperative effects, the energies E(c) as obtained from the periodic DFT calculations should exactly equal $\gamma_{\mathrm{HS}}\left(c\right)\Delta E_{\mathrm{HL}}$ for all configurations *c*. The values given in Table 1 are significantly larger for all mixed configurations ($0<\gamma_{\mathrm{HS}}<1$), independent of the density functional used, thus indicating the presence of cooperative effects. While the (unweighted) average values for Γ(c) are quite similar for LDA and PBE (8.3 vs. 8.8 kJ·mol${}^{-1}$, both more than twice as large as the experimental value of 3.0 kJ·mol${}^{-1}$ from Sinitskiy et al. [11]), the standard deviation of the values obtained from LDA is significantly larger than the one obtained from the PBE values (3.0 vs. 1.2 kJ·mol${}^{-1}$). In summary, the PBE calculations seem to fit much better to the SDM than the LDA calculations.

### 2.2. Ising-Like Model

Clearly, the sixteen different configurations of spin states that can be realized in a unit cell with only four SCO molecules are not representative of the configurations of larger lattices where the number of molecules is on the order of magnitude of Avogadro’s number. Obviously, in the case of ferromagnetic coupling, large clusters of equal spins are energetically favourable to configurations where neighbouring molecules have different spin states. For this reason, the results from the periodic DFT calculations were used to define coupling parameters for an Ising-like model with periodic boundary conditions, used to reproduce the thermal spin conversion of [Fe(phen)${}_{2}$(NCS)${}_{2}$]. In this model, each molecule is coupled to its twelve nearest neighbours in the same cell or in one of the surrounding 26 unit cells. That means, for instance, that the molecule at position I in Figure 1 is coupled to the nearest four molecules on position II, to the nearest four on position III and, finally, to the nearest four molecules on position IV. The complete coupling scheme is presented in Table 2.

The coupling parameters $J_{\mathrm{II}}$, $J_{\mathrm{III}}$, and $J_{\mathrm{IV}}$ (see Table 2) were determined by a fit procedure in a way that with the help of Equation (7) the energies E(c) of the sixteen configurations can be reproduced. The energies obtained in this way (last column in Table 1) deviate on average by about 3% from the PBE values. For the energy *B* (the generalized “external field”) in the Hamiltonian of the Ising-like model given by Equation (7), the energy difference $\Delta E_{\mathrm{HL}}\approx 10.3$ kJ·mol${}^{-1}$ obtained from the PBE calculations was taken. In order to exactly fit the experimental transition temperature, a total entropy difference of ΔS=58 J·mol${}^{-1}$ has been used, distinctly larger than the value of 49 J·mol${}^{-1}$ that was determined experimentally by Sorai and Saki [32], larger also than calculated values. If for ΔS a value obtained by first principles calculations [33,34,35,36] were taken, the transition temperature would have been shifted by more than 30 K, but the estimate for the interaction parameter Γ would not have been significantly affected.

**Figure 3:**
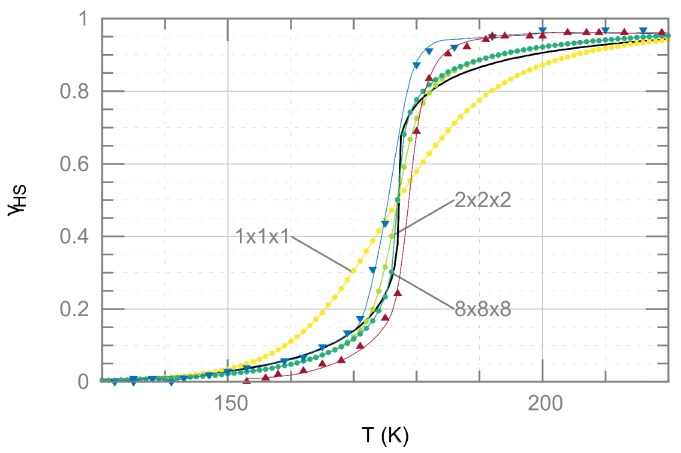
Measured and calculated temperature-dependent HS fraction $\gamma_{\mathrm{HS}}\left(T\right)$ of [Fe(phen)${}_{2}$(NCS)${}_{2}$]. Measured values which are extracted from susceptibility data from Gütlich et al. [37] (disregarding the residual HS fraction of about 17%) show a narrow hysteresis of about 2 K between the curves obtained upon heating and cooling (▵ and ∇, respectively, the thin lines are guides to the eyes). The thick black line represents the transition curve obtained from the Slichter–Drickamer model (SDM) by solving Equation (4) with Γ=3 kJ·mol${}^{-1}$, using a self consistent iterative procedure. The results of Monte Carlo (MC) simulations for supercells of different sizes as indicated are represented by filled circles.

MC simulations are used to calculate the temperature-dependent HS fraction $\gamma_{\mathrm{HS}}$ for Ising models encompassing $n^{3}$ unit cells, starting from n=1 (only one unit cell with four spins) up to a super cell with n=8 (512 unit cells with 2048 spins in total). Despite the couplings between the individual spins, the calculated transition curve for n=1 is rather gradual, not exhibiting strong cooperativity. MC simulations for a model with n=2 already yielded a much steeper transition curve, and the curves for n=3,…,8 are hardly distinguishable. Therefore, it is expected that a further increase of the lattice size *n* will not change the results. The curves for n=3,…,8 fit well to the transition curve obtained from the SDM with Γ=3 kJ·mol${}^{-1}$ (Figure 3). In contrast, at best reasonable agreement could be obtained between the simulated and the measured transition curve. Since the agreement between experiment and the SDM cannot be improved by increasing or decreasing Γ, one may conclude that an extension of the SDM (e.g., [38]) will be required in order to fit the experimental data. Concerning the results of the MC simulations for the Ising model, more realistic coupling parameters $J_{i,j}$ may be needed, which could be obtained from DFT calculations for larger cells.

**Figure 4:**
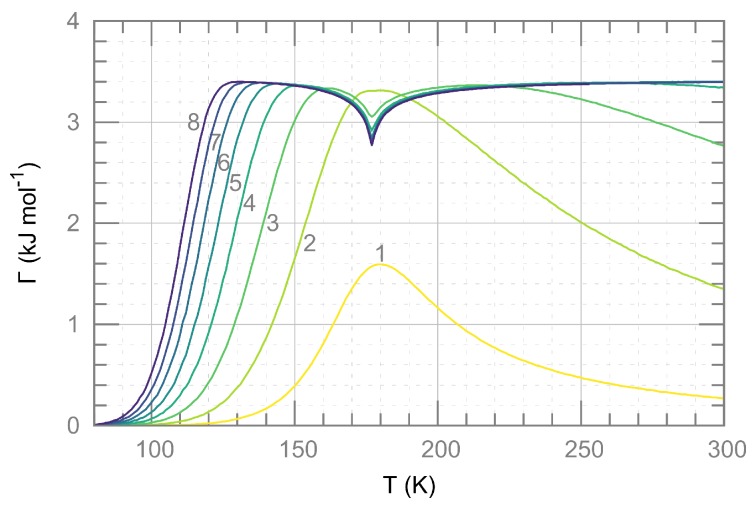
Temperature-dependent estimates for Γ obtained from a combination of enthalpies calculated with PBE (see Table 1) and MC simulations for supercells of size n×n×n with values of *n* (1 to 8) as indicated in the figure.

For each configuration of spin states that is reached in the course of the MC simulation, Γ(c) was calculated according to Equation (6). In this way, for each lattice size and each temperature, a thermal expectation value Γ(T) was calculated (Figure 4). For sufficiently large lattices, the expectation value for Γ(T) is almost independent of the temperature, with the remarkable exception of a small negative peak around the transition temperature, which might be due to the well-known effect of critical slowing down of MC calculations near phase transitions.

## 3. Computational Details

Periodic density functional calculations were performed with the software suite Quantum ESPRESSO [39]. Valence electrons were represented explicitly by plane waves with cutoffs of 75 Ry for the waves and 600 Ry for the charge density, and the interactions between these electrons were described within the generalized gradient approximation with the help of the exchange and correlation functional PBE from Perdew, Burke, and Ernzerhof [30]. Ultrasoft pseudopotentials [40] were employed [41] to treat the core electrons implicitly by replacing the all-electron Hamiltonian with a pseudo-Hamiltonian that describes the interactions between the ions and the valence electrons. The localization of the strongly correlated iron 3d electrons and the influence of London forces were described by the DFT+*U*+D2 model with the simplified scheme of Cococcioni et al. [42] together with a Hubbard *U* of 2.5 eV and the Grimme-D2 correction [43]. The Brillouin zone sampling was restricted to the Γ point. X-ray structures at T=15 K from Legrand et al. [29] were used as starting points for all geometry optimizations, and self-consistent wavefunctions and forces were converged to an accuracy of $10^{-7}$ and $10^{-5}$ atomic units, respectively.

Monte Carlo (MC) simulations for Ising-like models were performed with a home made program (available from the authors upon request) coded in Python 3 utilizing the Metropolis algorithm [44]. Cubic lattices with periodic boundary conditions were used, which consist of n×n×n cells with four different sites each (*n* varying from 1 to 8). Ising spins σ=±1 were assigned to all sites, with couplings between neighbouring spins as described in Section 2. To reach thermal equilibrium at a given temperature, $10^{5}$ sweeps were carried out, each sweep consisting out of *N* MC steps, where *N* denotes the number of lattice sites. During an individual MC step, the spin of a randomly chosen lattice site is flipped with a probability p(ΔE) that depends on the change of energy connected with the spin flip. This probability equals 1 if a spin flip would lower the energy (ΔE<0); otherwise, p(ΔE) equals b/(1+b), with *b* being the Boltzmann factor $\exp(-\Delta E/k_{B}T)$. After reaching thermal equilibration, an additional $10^{5}$ sweeps were carried out in order to calculate thermal expectation values (for the smaller lattices, 1×1×1 and 2×2×2, $10^{6}$ sweeps were carried out instead to gain better statistics). The MC simulations were performed for temperatures ranging from 0 to 300 K with a step size of 1 K.

## 4. Conclusions

At the example of the well-known spin crossover complex [Fe(phen)${}_{2}$(NCS)${}_{2}$], it has been demonstrated that the combination of periodic density functional calculations and Monte Carlo simulations for an Ising-like model allows a reasonable estimate for the phenomenological interaction parameter Γ to be obtained from first principles that describes the cooperativity of the spin transition within the Slichter–Drickamer model (the use of the term “first principles” in connection with DFT calculations is surely controversial, and this applies even more to DFT+*U* calculations, but one should keep in mind that the Hubbard *U* has to be determined only once for a large class of complexes with [Fe(II)N${}_{6}$] core and that Γ is not extremely sensitive to the choice of *U*). The agreement between the calculated and the experimentally-derived values for Γ is clearly better than in previous studies [11,12]. This may be due to the combination of electronic structure calculations (as compared to the empirical atom–atom potentials applied by Sinitskiy et al. [11]) and the Ising-like model (as compared to the restriction to a single unit cell in Reference [12]). However, to ensure that the result obtained for [Fe(phen)${}_{2}$(NCS)${}_{2}$] is not a lucky coincidence, it will be necessary to apply this method to a set of different spin crossover systems with varying degrees of cooperativity of the spin transition. DFT calculations for larger cells (containing at least twice the number of SCO molecules) could help to improve the simulated and measured transition curves. This might also bring one closer to an answer to the question that is naturally arising from these calculations: how can one rationalize the factors governing the cooperativity of the spin transition?

## Figures and Tables

**Table 1:** Calculated total electronic energies per molecule, E(c), Equation (5), and microscopic interaction parameters, Γ(c), Equation (6), in kJ·mol${}^{-1}$ for different spin configurations *c* as described in Section 2. The values derived from local density approximation (LDA) calculations are taken from Reference [12]. The last column presents energies obtained by an Ising-like model as described in Section 2. Symmetry equivalent configurations (e.g., nos. 2–5) are grouped in blocks.

No.	*c*	$\gamma_{\mathrm{HS}}$	LDA		PBE		Ising
			E(c)	Γ(c)	E(c)	Γ(c)	E(c)
1	LLLL	0.00	0.0		0.0		0.0
2	LHLL	0.25	4.4	10.4	4.3	9.2	4.3
3	LLHL	0.25	4.6	11.5	4.3	9.2	4.3
4	LLLH	0.25	4.6	11.5	4.3	9.2	4.3
5	HLLL	0.25	4.7	12.0	4.3	9.2	4.3
6	HLHL	0.50	6.2	5.2	6.8	6.6	6.8
7	LLHH	0.50	6.3	5.6	6.8	6.6	6.8
8	LHLH	0.50	7.0	8.4	7.5	9.4	7.5
9	HLLH	0.50	7.3	9.6	7.5	9.4	7.5
10	HHLL	0.50	7.6	10.8	7.8	10.6	7.8
11	LHHL	0.50	7.7	11.2	7.8	10.6	7.8
12	HLHH	0.75	8.2	4.5	9.3	8.4	9.4
13	HHHL	0.75	8.3	5.1	9.3	8.4	9.4
14	HHLH	0.75	8.3	5.1	9.3	8.4	9.4
15	LHHH	0.75	8.4	5.6	9.3	8.4	9.4
16	HHHH	1.00	9.8		10.3		10.2

**Table 2:** Coupling scheme for molecules at positions I to IV (as shown in Figure 1) with coupling parameters $J_{\mathrm{II}}=0.088$, $J_{\mathrm{III}}=0.213$, and $J_{\mathrm{IV}}=0.125$ (all given in kJ·mol${}^{-1}$).

	I	II	III	IV
I	–	$J_{\mathrm{II}}$	$J_{\mathrm{III}}$	$J_{\mathrm{IV}}$
II	$J_{\mathrm{II}}$	–	$J_{\mathrm{IV}}$	$J_{\mathrm{III}}$
III	$J_{\mathrm{III}}$	$J_{\mathrm{IV}}$	–	$J_{\mathrm{II}}$
IV	$J_{\mathrm{IV}}$	$J_{\mathrm{III}}$	$J_{\mathrm{II}}$	–
